# Alocanthedon, a new subgenus of *Chalicodoma* from Southeast Asia (Hymenoptera, Megachilidae)

**DOI:** 10.3897/zookeys.101.1182

**Published:** 2011-05-31

**Authors:** Michael S. Engel, Victor H. Gonzalez

**Affiliations:** 1 Division of Entomology, Natural History Museum, and Department of Ecology & Evolutionary Biology, 1501 Crestline Drive – Suite 140, University of Kansas, Lawrence, Kansas 66049–2811, USA; 2 Department of Ecology & Evolutionary Biology, 1200 Sunnyside Avenue, Haworth Hall, University of Kansas, Lawrence, Kansas 66045; Current address: USDA-ARS Bee Biology & Systematics Laboratory, Utah State University, Logan, Utah 84322–5310, USA

**Keywords:** Apoidea, Anthophila, Megachilini, *Chalicodoma*, taxonomy, Southeast Asia, Megachilinae, phylogeny

## Abstract

A new subgenus, *Alocanthedon* Engel and Gonzalez **subgen. n.**, is described for five species of unusual Southeast Asian bees in the genus *Chalicodoma* Lepeletier de Saint Fargeau (Megachilinae: Megachilini). The subgenus is most noteworthy for the deep postgenal depression or furrow in males (bordered outwardly near the base of the mandible by a protuberant, thick lamella) and the presence of a dense patch of black setae posteriorly in the forewing medial cell (except in one species) [resembling the dense patch of setae among the submarginal cells of *Thrinchostoma* Saussure (Halictidae: Halictinae: Halictini)]. The subgenus is characterized and distinguished from the related *Callomegachile* Michener. A key to the following five species presently included in the subgenus is provided: *Chalicodoma aterrimum* (Smith), *Chalicodoma atratiforme* (Meade-Waldo) **comb. n.**, *Chalicodoma memecylonae* Engel **sp. n.**, *Chalicodoma odontophorum* Engel **sp. n.**, and *Chalicodoma apoicola* Engel **sp. n.** *Chalicodoma (Callomegachile) atratiforme sininsulae* (Cockerell) is newly placed in synonymy with *C*. (*C*.) *fulvipenne* (Smith). Species have been collected from Memecylaceae (Myrtales) and Fabaceae (Fabales). The phylogenetic relationships of *Alocanthedon* among other Megachilini are briefly elaborated upon.

## Introduction

The Southeast Asian fauna of megachiline bees is particularly diverse but simultaneously poorly documented, underrepresented in collections, and with many species largely confused (especially in the diverse subgenus *Eutricharaea* Thomson of *Megachile* Latreille). In recent years several rather remarkable groups of species have been identified among this fauna (Baker and Engel 2006; Engel and Baker 2006). The purpose of the present contribution is to put on record yet another new group of peculiar Southeast Asian megachiline bees so that their names may be available for forthcoming works on the phylogeny and classification of the tribe and to bring them to the attention of melittologists working with this fauna. Herein, we propose a new subgeneric name, *Alocanthedon* Engel and Gonzalez subgen. n., for five unusual species in the Old World genus *Chalicodoma* Lepeletier de Saint Fargeau (Megachilinae: Megachilini) that are easily recognized by a unique combination of morphological characters in both sexes, especially in the male. Unlike all other *Chalicodoma*, males in the new subgenus have a deep postgenal depression or furrow bordered outwardly by a protuberant, thick lamella and a dense patch of black setae posteriorly in the forewing medial cell (except in one species). To date, the only other group of bees known to have a similar patch of black setae on the forewing is the sweat bee genus *Thrinchostoma* Saussure (Halictidae: Halictinae: Halictini). However, such a patch of setae is found among the submarginal cells of the forewing in both sexes of *Thrinchostoma*.

Following the classificatory proposal of Gonzalez (2008), *Chalicodoma* is herein recognized in a narrower sense than that of (Michener (1962, 1965). As here understood, it includes all subgenera of Group 2 of *Megachile* s.l. *sensu* Michener (2007), except for *Matangapis* Baker and Engel and those taxa with heriadiform or hoplitiform bodies with sparse pubescence (i.e., *Chelostomoda* Michener and related groups). The relationships among the seven subgenera presently recognized in *Chalicodoma* (*Callomegachile* Michener, *Cestella* Pasteels, *Chalicodoma* s.str., *Cuspidella* Pasteels, *Gronoceras* Cockerell, *Largella* Pasteels, and *Pseudomegachile* Friese) need to be studied in detail. Such subgenera are highly diverse, morphologically heterogeneous, and as distinct as many genera of bees. Future work may show that generic status might be warranted for some or all.

We also briefly discuss the phylogenetic relationships of *Alocanthedon* among the subgenera of *Chalicodoma* and provide an overview of the morphological diversity of the related *Callomegachile* as well as taxonomic notes on other rare Southeast Asian species.

## Materials and Methods

Morphological terminology follows that of Engel (2001) and Michener (2007) while the format for the descriptions loosely follows those of Michener (1965), Baker and Engel (2006), Engel and Baker (2006), and Gonzalez et al. (2010) for megachiline bees. Institutional acronyms used herein are: SEMC, Snow Entomological Collection, Division of Entomology, University of Kansas Natural History Museum, Lawrence, Kansas, USA; NHML, Department of Entomology, The Natural History Museum, London, UK; and NSMT, National Science Museum (Natural History Museum), Tokyo, Japan.

To explore the phylogenetic relationships of these rare bees, we used the morphological characters and data set of Gonzalez (2008) for the phylogeny of Megachilini (six outgroup species, 107 ingroup species, and 231 characters). This data set is available from the authors or can be obtained from the unpublished dissertation, which is freely accessed throughout the University of Kansas libraries (http://kuscholarworks.ku.edu/dspace/handle/1808/4187). Because the specimens were not dissected, 31 characters, corresponding to some traits of the labiomaxillary complex, sixth sternum of females, sting apparatus, and male hidden sterna could not be coded. Parsimony analyses were performed in an Intel® CoreTM i3 processor using Tree analysis using New Technology (TNT; Goloboff et al. 2003). All characters were treated as unweighted, unordered, and nonadditive to allow characters to reverse freely and examine possible hypotheses of evolutionary relationships. Tree search in TNT was done by implementing sectorial searches (SS) with tree drifting (TD) and tree fusing (TF) and ratchet runs with TD and TF. We used the following search: keep a maximum of 10000 random trees, 500 random addition sequences, and 1000 ratchet iterations, including 100 cycles of TD and 100 rounds of TF per iteration. Trees were visualized and printed using Winclada (Nixon 1999).

## Systematics

**Tribe Megachilini Latreille**

**Genus** *Chalicodoma* **Lepeletier de Saint Fargeau**

### Alocanthedon

Engel and Gonzalez subgen. n.

urn:lsid:zoobank.org:act:42B75DAA-6A32-4AAF-8209-4E1BD5E56B22

http://species-id.net/wiki/Alocanthedon

#### Type species.

*Chalicodoma (Alocanthedon) odontophorum* Engel, sp. n.

#### Diagnosisn.

**Figures 1–6.**
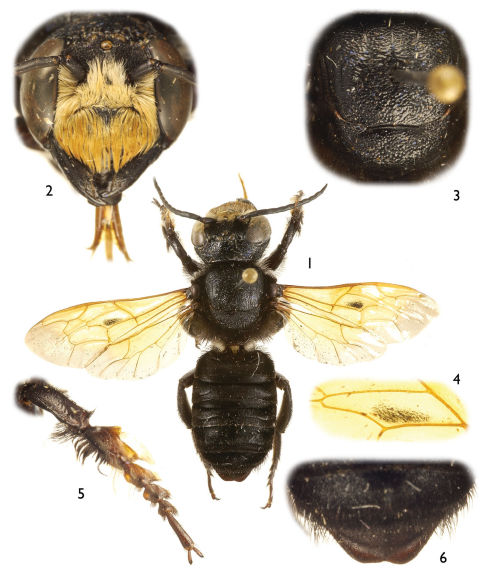
Photomicrographs of male of *Chalicodoma* (*Alocanthedon*) *odontophorum* Engel, sp. n. **1** Dorsal habitus **2** Facial aspect **3** Dorsal aspect of mesoscutum and mesoscutellum **4** Detail of forewing medial cell **5** Protarsus, pro-pretarsus, and protibia **6** Dorsal aspect of metasomal terga V and VI.

**Figures 7–9.**
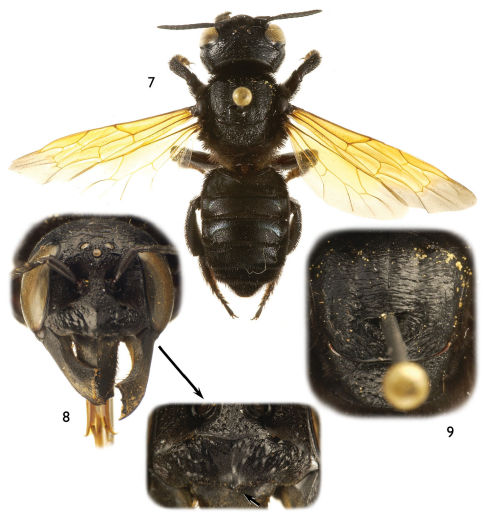
Photomicrographs of female of *Chalicodoma* (*Alocanthedon*) *odontophorum* Engel, sp. n. **7** Dorsal habitus **8** Facial aspect (with expanded detail of clypeus, arrow indicating medioapical tubercle) **9** Dorsal aspect of mesoscutum and mesoscutellum.

**Figures 10–14.**
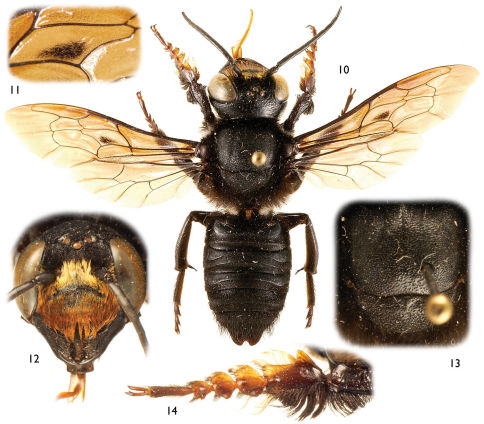
Photomicrographs of male of *Chalicodoma* (*Alocanthedon*) *apoicola* Engel, sp. n. **10** Dorsal habitus **11** Detail of forewing medial cell **12** Facial aspect **13** Dorsal aspect of mesoscutum and mesoscutellum **14** Protarsus, pro-pretarsus, and apex of protibia.

**Figures 15–16.**
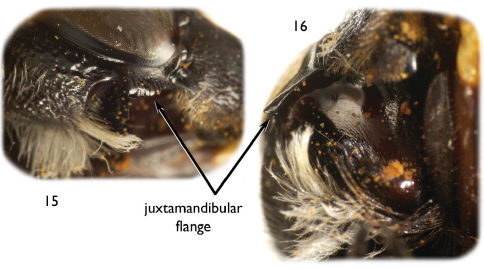
Photomicrographs of male head of *Chalicodoma* (*Alocanthedon*) *apoicola* Engel, sp. n. depicting juxtamandibular flange/lamella and postgenal depression **15** Right lateral aspect of apical portion of head **16** Ventral aspect of right side of head.

**Figures 17–22.**
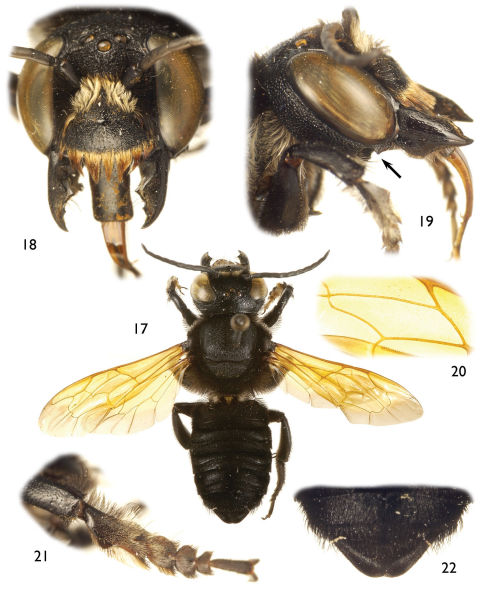
Photomicrographs of male of *Chalicodoma* (*Alocanthedon*) *memecylonae* Engel, sp. n. **17** Dorsal habitus **18** Facial aspect **19** Lateral aspect of head, arrow indicates postgenal flange, note also broad, medial inferior protuberance of male mandible **20** Detail of forewing medial cell **21** Protarsus, pro-pretarsus, and apex of protibia **22** Dorsal aspect of metasomal terga V and VI.

**Figures 23–25.**
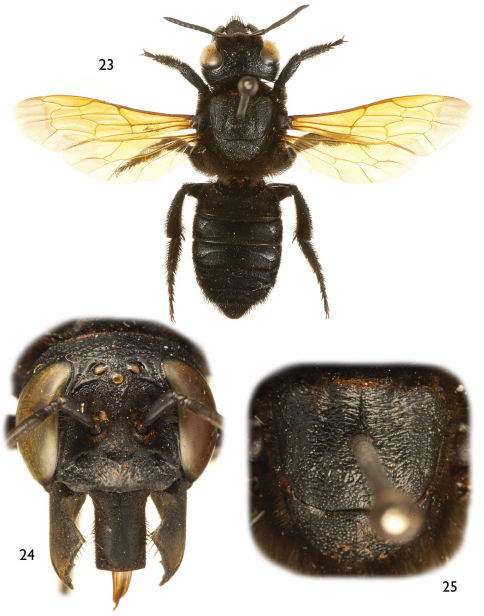
Photomicrographs of female of *Chalicodoma* (*Alocanthedon*) *memecylonae* Engel, sp. n. **23** Dorsal habitus **24** Facial aspect **25** Dorsal aspect of mesoscutum and mesoscutellum.

**Figures 26–34.**
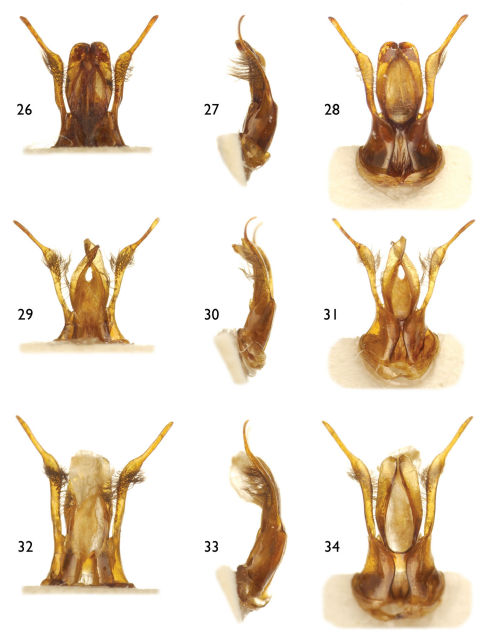
Photomicrographs of representative male genitalia for species of *Alocanthedon* Engel and Gonzalez, subgen. n. in ventral, lateral and dorsal aspects **26–28** *Chalicodoma* (*Alocanthedon*) *odontophorum* Engel, sp. n. **29–31** *C*. (*A*.) *memecylonae* Engel, sp. n. **32–34** *C*. (*A*.) *atratiforme* (Meade-Waldo).

**Figures 35–40.**
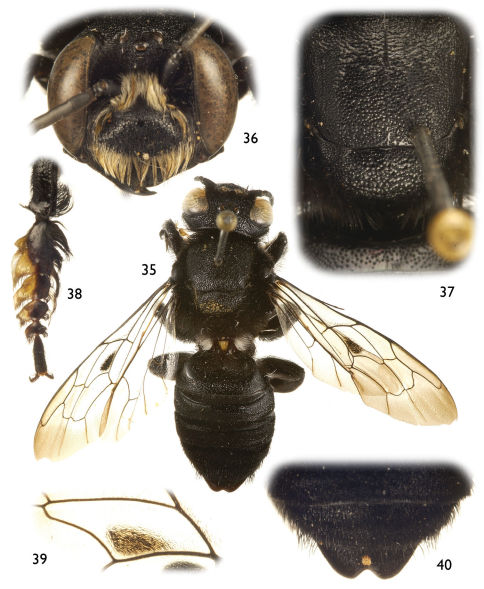
Photomicrographs of male of *Chalicodoma* (*Alocanthedon*) *aterrimum* (Smith) **35** Dorsal habitus **36** Facial aspect **37** Dorsal aspect of mesoscutum and mesoscutellum (small portion of first metasomal tergum also visible) **38** Protarsus, pro-pretarsus, and apex of protibia **39** Detail of forewing medial cell **40** Dorsal aspect of metasomal terga V, VI, and apical portion of IV.

**Figures 41–43.**
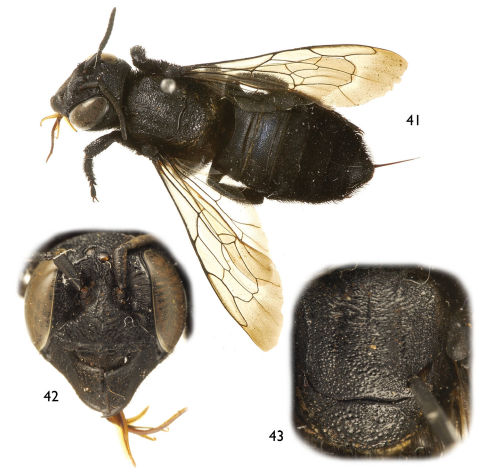
Photomicrographs of female of *Chalicodoma* (*Alocanthedon*) *aterrimum* (Smith) **41** Dorsal habitus **42** Facial aspect **43** Dorsal aspect of mesoscutum and mesoscutellum.

**Figures 44–46.**
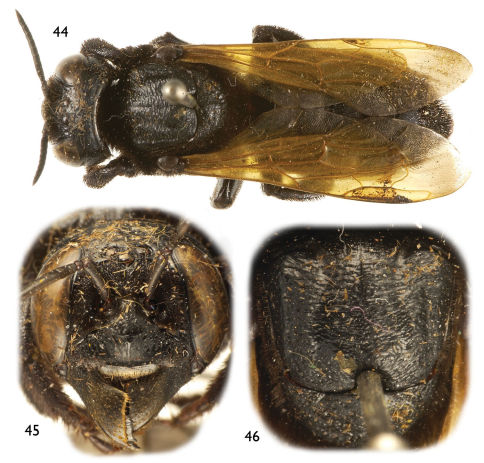
Photomicrographs of female of *Chalicodoma* (*Alocanthedon*) *atratiforme* (Meade-Waldo), comb. n. **44** Dorsal habitus **45** Facial aspect **46** Dorsal aspect of mesoscutum and mesoscutellum.

Large (ca. 20–25 mm), black, parallel-sided megachilines resembling some large, black species of *Callomegachile* such as those of the Eumegachilana group (Figs. 1, 7, 10, 17, 23, 35, 41, 44) but in males with juxtamandibular flange or lamella and deep postgenal depression (Figs. 15, 16, 19); with oblique carina or lamella medially on disc of procoxa; with modified pro- (Figs. 5, 14, 21, 38) and mesotarsi; with dense cluster of short, black setae forming a conspicuous spot in the posterior half of the forewing medial cell (Figs. 4, 11, 39, except in *Chalicodoma memecylonae*); with five exposed metasomal sterna and with apical margin of sternum six typically exposed; with basally large gonocoxae (in comparison with those of the large species of Eumegachilana group where they are disproportionately small for the size of the bee) that are attenuate, divergent, upcurved at apices, greatly exceeding apices of penis valves, and without setae apically (Figs. 26–34); with penis valves slightly expanded apically; and in females with broad, not porrect, 4-toothed mandibles, with the outer surface dull, minutely roughened, and punctate; with clypeus slightly concave to V-shaped epistomal sulcus basally; with the pretarsal claws simple, basally with short, stout seta; and with the metasoma parallel-sided.

#### Description.

*Male*: Mandible tridentate, with median inferior swelling or protuberance, basal projection absent; torulus with distinct lamella on upper half of inner margin; first flagellomere wider than long, length much less than one-half length of second flagellomere. Preoccipital carina distinct, continuing from vertex to gena; postgena bordering hypostoma with deep depression, posteriorly bordered by dense brush of black setae and long, finer patch of white setae, depression with bordering juxtamandibular flange or lamella near anterior mandibular base and bordering compound eye (Figs. 15, 16). Forewing with dense cluster of short, black, simple, lanceolate setae forming a conspicuous spot in posterior half of medial cell (Figs. 4, 11, 39: except in *Chalicodoma memecylonae*, Fig. 20). Pronotal lobe with strong carina; procoxa with apical, anteriorly-directed spine, with oblique carina or lamella medially on procoxal surface, anterior surface without rufescent bristles; protarsus greatly modified, flattened and expanded in species-specific forms, with variegated fringes; meso- and metafemora slightly swollen; mesotibial spur present; mesotarsi flattened with concave inner basal surfaces, posterior border variously modified; pretarsal claws symmetrical, cleft; mesobasitarsus weakly to strongly arched basally, with variously developed basal ventral concavity; metatarsi unmodified, slender; metasomal tergum VI with preapical carina gently concave medially (depth of concavity varies dramatically across species), without teeth; metasomal sternum V exposed, densely pubescent; apex of metasomal sternum VI normally exposed, densely pubescent; gonocoxae relatively large (by comparison with those of the large species of Eumegachilana group where they are disproportionately small for the size of the bee), attenuate, divergent, apices upcurved, greatly exceeding apices of penis valves, without setae apically (Figs. 26–34); volsella pointed, articulate, distinguished as separated sclerite; penis valves slightly expanded apically.

*Female*: Mandible broad, not porrect or elongate (similar to *Callomegachile*) except somewhat elongate in *Chalicodoma odontophorum*, 4-toothed (third tooth reduced in *Chalicodoma aterrimum*), without cutting edge, outer surface dull, minutely roughened and coarsely and shallowly punctate. Clypeus not protuberant, not covering labral base; first flagellomere wider than long, about one-half length of second flagellomere. Pretarsal claws simple, symmetrical, basally with short, stout seta. Metasoma parallel-sided; tergum VI very weakly concave in profile, with pubescence as on preceding terga; sternum VI with scopal setae as on preceding sterna, without bare rim; sterna without apical pubescent bands.

#### Etymology.

The new genus-group name is a combination of the Greek words *alokos* (meaning, “furrow”) and *anthedon* (meaning, “bee”), and is a reference to the deep postgenal furrow universally in males of this lineage. The name is feminine.

#### Included species.

In addition to the type species the subgenus includes the following taxa: *Chalicodoma aterrimum* (Smith 1862), *Chalicodoma atratiforme* (Meade-Waldo, 1914), *Chalicodoma memecylonae* Engel sp. n., and *Chalicodoma apoicola* Engel sp. n. (Table 1).

#### Comments.

The five currently included species are superficially quite similar, but for the hyaline or dark fuscous, rather than yellow, wings in *Chalicodoma aterrimum* and *Chalicodoma apoicola*, accordingly the new species are described in reference to the type species rather than repeat largely identical blocks of text. Owing the presence of species of *Alocanthedon* in the Philippines as well as across Wallace’s Line in Sulawesi it is likely that as of yet unrecognized taxa for the subgenus may occur in places throughout Indonesia and Malaysian Borneo, and perhaps as far East as Irian Jaya. Additional collecting of bees, for all groups, is needed across all of these islands.

#### Chalicodoma (Alocanthedon) odontophorum

Engel sp. n.

urn:lsid:zoobank.org:act:1DA0608B-A874-4338-BDAE-FECE7D9E7FEA

http://species-id.net/wiki/Chalicodoma_(Alocanthedon)_odontophorum

Figs 1 9 26–28 

##### Holotype.

Thailand: ♂, Sakaerat DDF [Nakhon Ratchasima Province, Sakaerat Environmental Research Area, ca. 40 km South Nakhon Ratchasima], 20 June 1995 (SEMC).

##### Paratypes.

Thailand: ♀, Sakaerat DDF [Nakhon Ratchasima Province, Sakaerat Environmental Research Area, ca. 40 km South Nakhon Ratchasima], 17 June 1995 (SEMC); 1♂, Siam (SEMC).

Myanmar: 1♀, Middle Tenasserim, Thaungyin Valley, 5.93 [May 1893], C.T. Bingham (NHML).

##### Diagnosis.

Both sexes of this species have yellow forewings with grayish hyaline apex. The male can be easily distinguished by the clypeus densely covered by long, appressed, apically-directed setae obscuring integument (Fig. 2) and the shape of the modified protarsi (Fig. 5). The female of this species is recognized by the clypeus with a pronounced, erect, medioapical tubercle (Fig. 8), the elongate mandibles and the labrum, with apical margin medially convex and apical fringe of erect setae separated from labral apical margin by at least one median ocellar diameter or slightly more.

##### Description.

As for the subgenus with the following additions: *Male*: Total body length 20 mm; forewing length 13.3 mm. Head broader than long (width 5.3 mm, length 4.0 mm); inner orbits of compound eyes slightly divergent below; intertorular distance 1.6 times torulorbital distance; interocellar distance 1.8 times median ocellar diameter, slightly shorter than ocellocular distance; ocelloccipital distance 4.2 times median ocellar diameter; compound eye about 2.2 times longer than wide, 1.2 times wider than gena in profile. Mandible with three teeth, with prominent, broad median inferior protuberance bearing dense, short, black setae. Juxtamandibular flange about twice as long as posterior height. Labrum rectangular, with apical row of stiff, erect, long setae. Clypeus broad, width more than three times medial length. Scape length about 2.5 times width; first flagellomere short, nearly one-third length of second flagellomere; remaining flagellomeres all much longer than wide, apicalmost flagellomere with broadly rounded apex, not tapering. Mesoscutum with distinct notauli and parapsidal lines. Procoxal spine elongate, with weak depression between spine and medial, transverse carina of procoxa, posterobasally setose on spine, anterior surface not setose; protibia with strong, outer, posterior carina running along apical three-quarters of length, apically produced into small posteriorly-directed spine before continuing transversely across apex to outer, anterior border where it forms definite ridge but not carinate, apical anterior surface faintly depressed in profile view; protarsus modified as in figure 5; meso- and metafemora somewhat swollen; mesotibial spur curved, with bluntly rounded apex; mesobasitarsus with inner surface concave basally, posterior border along concavity relatively straight; outer metatibial spur blunt at apex (not tapering to acute apex); pretarsal claws long, curved, apically cleft. Postgradular depressions deeper than in female; terga II–V with apical transverse ridge (caudad postgradular depression), somewhat sinuate laterally, weak medially on terga II–IV; preapical carina of sixth tergum produced, weakly and broadly concave medially (Fig. 6). Genitalia as in figures 26–28.

Integument black throughout except tegula, legs, and metasomal sterna largely dark reddish brown (nearly black in many areas), and expansion of protarsi more translucent brown. Wings orange-yellow except apical margin of forewing and apical and posterior margins of hind wing grayish hyaline (Fig. 1); venation ferruginous to orange-yellow.

Mandible with outer surface dull, irregularly punctate and microreticulate; labrum strongly imbricate and impunctate; clypeus with small, contiguous punctures, with thin mediolongitudinal impunctate area; supraclypeal area and face below ocelli with small, contiguous punctures, punctures becoming more irregular at level of median ocellus; area between ocelli with small, contiguous punctures; ocellocular area with somewhat larger, coarser punctures separated by areas of finely imbricate integument; vertex with coarse, shallow punctures separated by a puncture width or less, integument between finely imbricate, punctures becoming more shallow and faint toward preoccipital carina; upper gena with irregular, elongate punctures separated by finely imbricate integument, remainder of gena and posterior postgena with more regular punctures separated by a puncture width or less, integument otherwise finely imbricate; postgenal surface inside of deep postgenal depression with scattered minute punctures separated by faintly imbricate to smooth integument; outer surface of juxtamandibular lamella with irregular punctures and imbricate integument. Pronotum imbricate, with small punctures separated by a puncture width or less; mesoscutum anteriorly and medially transversely wrinkled with irregular punctures, such integument blending laterally outside of parapsidal lines and posteriorly to coarsely punctate, punctures separated by a puncture width or less, those outside of parapsidal lines somewhat smaller and more regularly defined than those posteriorly, integument between punctures finely imbricate; tegula finely imbricate and minutely punctate, punctures separated by less than a puncture width, except along outer rim impunctate; axillae and mesoscutellum coarsely and contiguously punctate except mediobasally on mesoscutellum with punctures smaller and gradually becoming separated by a puncture width or less; metanotum imbricate with small punctures separated by a puncture width or less; pleura coarsely and contiguously punctate, those punctures along omaular ridge and ventrally somewhat dorsoventrally elongate, giving ventral surface a somewhat dorsoventrally rugulose appearance; declivitous basal area of propodeum with single row of coarse alveolae along extreme basal border, row interrupted medially, otherwise surface imbricate and impunctate; lateral surface imbricate with small punctures separated by less than a puncture width, gradually becoming more widely spaced posteriorly and on posterior surface. Anterior-facing surface of first metasomal tergum finely imbricate, dorsal-facing surface imbricate with small punctures separated by a puncture width or less, nearly contiguous in most areas; remaining terga sculptured as on dorsal-facing surface of first metasomal tergum; terga with narrow impunctate apical rims; sterna smooth to finely imbricate, with small punctures separated by less than a puncture width.

Pubescence generally dark fuscous to black except as follows: clypeus, supraclypeal area, and face outside of antennal toruli with dense, long, minutely-branched, tawny to white setae, largely obscuring the integument, those on clypeus more strongly tawny and largely appressed and apically directed; thin fringe of short, fine, silvery white setae along outer border of patch of black setae on median inferior protuberance and running proximally to mandibular condyle; dense patch of white pubescence immediately posterior to dense patch of somewhat shorter black setae at posterior end of postgenal depression, white patch largely occupying area at meeting of postgena and ventral area of gena; white, long setae on pronotal lateral surfaces, outer borders of propleura, outer base of procoxa, and preomaular area; white and black setae arranged on protarsus and apex of protibia as in figure 5; and white setae on propodeal lateral surface, most dense ventrally, near metacoxa; wing setae generally yellow or tawny yellow except dense cluster of short, black setae forming a conspicuous spot in posterior half of forewing medial cell (Fig. 4).

*Female*: As for the male except in usual sexual differences and as follows: Total body length 19–21 mm; forewing length 14.9 mm. Head broader than long (width 6.1 mm, length 4.5 mm); intertorular distance about as long as torulorbital distance; interocellar distance 2.4 times median ocellar diameter, 0.8 times ocellocular distance; ocelloccipital distance 6.9 times median ocellar diameter; compound eye about twice as long as wide, slightly narrower than gena in profile. Mandible with four teeth; body of mandible elongate, basal section distinctly longer than apical, dentate margin (Fig. 8). Labrum rectangular, with apical row of stiff, erect, long setae separated from apical margin by at least one median ocellar diameter; apical margin medially convex. Clypeus with pronounced, medioapical tubercle (all species have a minute point here, but not distinctly tuberculate as in this species) (Fig. 8). Scape length more than three times width; first flagellomere short, about one-half length of second flagellomere; remaining flagellomeres all about twice as long as wide. Procoxae, tibiae, tarsi, and spurs unmodified; pretarsal claws long, curved, simple. Postgradular depressions faint; terga without transverse ridges, with slight lateral swellings on terga II and III.

Clypeus imbricate with coarse, shallow punctures separated by less than a puncture width in basal half except medially such punctures restricted to basal border; supraclypeal area with smaller coarse punctures than those on clypeus, punctures separated by less than a puncture width; face below ocelli with small, contiguous punctures, punctures becoming more irregular at level of median ocellus; ocellocular area with somewhat larger, coarser punctures separated by areas of finely imbricate integument; vertex with coarse, shallow punctures separated by 0.5–1.5 times a puncture width, integument between finely imbricate, punctures becoming more shallow and faint toward preoccipital carina; postgena strongly rugulose; hypostoma imbricate. Mesoscutum anteriorly and medially transversely wrinkled, more weakly so than in male, with irregular punctures, such integument blending laterally and posteriorly to faintly coarsely punctate, punctures separated by less than a puncture width, those outside of parapsidal lines sparse, integument between punctures finely imbricate; axillae and mesoscutellum strongly coarsely and contiguously punctate except mediobasally on mesoscutellum with punctures smaller and gradually becoming separated by a puncture width or less. Dorsal-facing surface of first metasomal tergum imbricate with small punctures separated by a puncture width or less medially and nearly contiguous laterally; remaining terga sculptured as on lateral areas of dorsal-facing surface of first metasomal tergum.

Usual sex differences in setation; pubescence dark fuscous to black except microtrichia on inner surface of mandible dark golden and small dirty white patch on lateral surface of propodeum near metacoxa; clypeus and supraclypeal area not obscured by dense pubescence; ventral surfaces of mesepisternum, coxae, trochanters, base of femora, anterior margins of metatibia and metafemur, and sternal scopa with capitate setae.

**Table 1.** Summary of currently included species in subgenus *Alocanthedon*.

Species	General distribution
*Chalicodoma apoicola* Engel sp. n.	Philippines: Mindanao: Davao del Sur
*Chalicodoma aterrimum* (Smith, 1862)	Sulawesi
*Chalicodoma atratiforme* (Meade-Waldo, 1914), comb. n.	Myanmar: Tanintharyi Division (= Tenasserim)
	Thailand: Uthai Thani Province
	Malaysia: Pahang State, Negeri Sembilan State
*Chalicodoma memecylonae* Engel sp. n.	Malaysia: Penang State, Perak State, Selangor State, Pahang State
*Chalicodoma odontophorum* Engel sp. n.	Thailand: Nakhon Ratchasima Province
	Myanmar: Tanintharyi Division

##### Etymology.

The specific epithet is a combination of the Greek word *odontos* (meaning, “teeth”) and suffix –*phor* (meaning, “carry”).

##### Floral records.

The holotype and paratype from Sakaerat were captured at flowers of *Sindora siamensis* Teijsman & Miquel (Fabales: Fabaceae: Caesalpinioideae: Detarieae).

#### Chalicodoma (Alocanthedon) apoicola

Engel sp. n.

urn:lsid:zoobank.org:act:A7C422CE-C4EB-4C1A-A8B4-541DB0B1E39C

http://species-id.net/wiki/Chalicodoma_(Alocanthedon)_apoicola

Figs 10 16 

##### Holotype.

Philippines: ♂, Mindanao, Tagurano [Davao del Sur, near Mt. Apo and Mt. Apo National Park], Davao City, 25–26.vi.1977 [25–26 June 1977], Y. Kurosawa (NSMT).

##### Diagnosisn.

The new species is most similar to *Chalicodoma aterrimum*, but can be distinguished from this and other *Alocanthedon* by the following combination of traits: clypeus covered with dense, long, appressed, reddish setae obscuring integument (Fig. 12); forewing dark fuscous with black venation; dense black setal patch present posteriorly in forewing medial cell (Fig. 11); dorsal-facing surface of first metasomal tergum with large, anterobasal areas of impunctate and imbricate integument; terga with strong transverse ridges on non-depressed, postgradular discs, carinate on terga II and III; sixth metasomal tergum deeply concave medioapically; medioapical apical margin of second metasomal sternum convex as short, broad extension; protibial apical outer surface distinctly depressed; outer anterior margin of protibia with dense fringe of long black setae; and unique protarsal shape and setation (Fig. 14).

##### Description.

As described for *Chalicodoma odontophorum* (*vide supra*) except as follows: *Male*: Total body length 24.6 mm; forewing length 19.3 mm. Head broader than long (width 7.4 mm, length 5.5 mm); intertorular distance 1.9 times torulorbital distance; interocellar distance 1.5 times median ocellar width, 1.1 times ocellocular distance; ocelloccipital distance 3.5 times median ocellar width; compound eye about twice as long as wide, about as wide as gena in profile. Protibia with outer posterior carina running along apical three-quarters of length, apically produced into small posteriorly-directed spine before continuing transversely across apex to outer, anterior border where it forms a carinate ridge for short distance along depression, apical anterior surface distinctly depressed; protarsus modified as in figure 14; mesotibial spur relatively straight; mesobasitarsus with inner surface deeply concave basally, posterior border along concavity notched, such that there is a posterior protuberance bordering the concavity. Metasomal terga II–V with apical transverse ridge (caudad postgradular depression), somewhat sinuate laterally, distinctly carinate on terga II–III except medially, strongly ridged on tergum IV, weak on tergum V; carina of sixth tergum produced, medioapical margin of carina of sixth metasomal tergum strongly and deeply concave (Fig. 10).

Wings dark fuscous, infumate (Fig. 10); venation black.

Mesoscutum anteriorly and medially transversely somewhat wrinkled (not as strongly so as in *Chalicodoma odontophorum*) with irregular punctures, such integument blending laterally outside of parapsidal lines and posteriorly to coarsely punctate, punctures separated by less than a puncture width, nearly contiguous in many areas, those punctures outside of parapsidal lines somewhat smaller and more regularly defined than those posteriorly, integument between punctures finely imbricate; lateral surface of propodeum imbricate with small punctures separated by a puncture width or less, gradually becoming more widely spaced posteriorly and on posterior surface. Dorsal-facing surface of first metasomal tergum imbricate with small punctures separated by a puncture width or less, nearly contiguous in most areas, with large laterobasal areas of impunctate (and asetose) and more distinctly imbricate integument; remaining terga sculptured as on dorsal-facing surface of first metasomal tergum, although punctures typically more tightly packed.

Pubescence generally dark fuscous to black except as follows: clypeus and face outside of antennal toruli with dense, long, minutely-branched, reddish setae, largely obscuring the integument, those on clypeus more strongly reddish and largely appressed and apically directed; supraclypeal area with similar setae to those on face except more tawny in color (Fig. 12); wing setae generally fine and black, dense cluster of short, black setae forming a conspicuous spot in posterior half of forewing medial cell (Fig. 11).

*Female*: Unknown.

##### Etymology.

The specific epithet is a combination of Mount Apo and the Latin suffix –*cola*, meaning “dweller”. The name is treated as a noun in apposition.

##### Comments.

Permission to dissect the holotype and only known specimen was not provided and thus the genitalia remain unknown for this distinctive species. Nonetheless, genitalic variation is relatively minor across species of *Alocanthedon* and the structure of the head, tarsi, metasomal terga and sterna, and integumental sculpturing will sufficiently serve to identify future material of this species.

*Chalicodoma apoicola*, herein described from the male alone, occurs in the same region as *Callomegachile (Callomegachile) davaonensis* (Cockerell 1918), described from the female sex and from a nearby area. Since the most distinguishing features of the subgenus are in the male it is possible that the latter species belongs to *Alocanthedon* and may be closely allied to *Chalicodoma apoicola*. It is tempting to speculate that *Chalicodoma apoicola* represents the unknown male for *Chalicodoma davaonensis*. From the original description, however, this seems unlikely given the significantly larger size of *Capoicola* (*ca*. 15 mm in *Chalicodoma davaonensis*, total length smaller than the forewing length in *Chalicodoma apoicola*) and the reddish translucent wings and ferruginous pterostigma and veins of *Chalicodoma davaonensis* (in this regard more similar to some of the Malaysian, Thai, and Burmese species of *Alocanthedon*). As already noted, significant collecting efforts for bees in Mindanao are needed so as to more accurately characterize these species and to more fully understand the fauna.

#### Chalicodoma (Alocanthedon) memecylonae

Engel sp. n.

urn:lsid:zoobank.org:act:066C973E-81A4-496A-BA57-F7701C49DC33

http://species-id.net/wiki/Chalicodoma_(Alocanthedon)_memecylonae

Figs 17 25 29–31 

##### Holotype.

Malaysia (Peninsular): ♂, Malaya, Penang, Batu Feringgi, 17 November 1963, H.T. Pagden (NHML).

##### Paratypes.

Malaysia (Peninsular): 2♂♂, Malaya, Pangkor Island, 5.i.1958 [5 January 1958], H.T. Pagden (NHML); 1♂, Malaya, Penang, Botanical Gardens at flowers *Duranta* [an introduced ornamental of Verbenaceae, native to the Americas, and accordingly not considered a host plant record herein], 11.xi.1958 [11 November 1958], H.T. Pagden (NHML); 1♂, Malaya, Penang, Batu Feringgi, 17 November 1963, H.T. Pagden (NHML); 1♀, Malaya, Penang, Mt. Erskine Road, at *Memecylon*, 18 July 1955, H.T. Pagden (NHML); 1♀, Malaya, Penang, Mt. Erskine Road, 3 June 1955, H.T. Pagden (NHML); 1♀, Malaya, Penang, Mt. Erskine Road, 9 August 1955, H.T. Pagden (SEMC); 1♀, Malaya, Kuala Lumpur, 10.9.1933 [10 September 1933], H.M. Pendlebury, Ex F.M.S. [Federated Malay States] Museum (NHML); 1♀, Malay Penin. [Peninsula], West Coast, Langkawi Is. [Island], 19 April 1928, H.M. Pendlebury, Ex F.M.S. [Federated Malay States] Museum (NHML); 1♀, Selangor, Serdang, 10.xii.1928 [10 December 1928], H.T. Pagden (NHML); 1♀, Serdang, in Memecy. [*Memecylon*], 10.xii.1928 [10 December 1928], H.T. Pagden (NHML).

##### Diagnosisn.

Both sexes of this species have yellow forewings with grayish hyaline apex. The male can be easily recognized by the clypeus with the disc not densely covered by setae (Fig. 18) and the absence of a patch of dense setae in the medial cell of the forewing (Fig. 20). The female is most similar to *Chalicodoma atratiforme* from which it can be separated by the mesoscutum with more pronounced transverse wrinkling on disc, posteriorly with well-defined coarse, irregular punctures separated by a less than a puncture width, not loosely arranged in transverse series like those in wrinkles (Fig. 25). Also, the hypostomal area is more coarsely punctate than in *Chalicodoma atratiforme*.

##### Description.

As described for *Chalicodoma odontophorum* (*vide supra*) except as follows: Total body length 18.0 mm; forewing length 12.7 mm. Head broader than long (width 5.0 mm, length 3.7 mm); intertorular distance 1.6 times torulorbital distance; interocellar distance 1.9 times median ocellar diameter, 1.2 times ocellocular distance; ocelloccipital distance 3.4 times median ocellar diameter; compound eye about twice as long as wide, slightly broader than gena in profile. Procoxal spine shorter and broader than that of *Chalicodoma odontophorum*, with weak depression between spine and oblique lamella of procoxa, lamella short, posterobasally setose on spine, anterior surface not setose; protibia with strong, outer, posterior carina running along apical three-quarters of length, apically produced into small posteriorly-directed spine, not carinate along transverse apex of protibia, anterior border ridged but not carinate, apical anterior surface not depressed; protarsus modified as in figure 21; meso- and metafemora somewhat swollen; mesotibial spur relatively straight, with bluntly rounded apex. Postgradular depressions deeper and broader than in female; terga II–IV with apical transverse ridge (caudad postgradular depression), ridges somewhat sinuate laterally, weak medially on terga II–III, entirely weak on tergum IV; preapical carina of sixth tergum produced, weakly and narrowly concave medially (Fig. 22). Genitalia as in figures 29–31.

Integument black throughout except tegula, legs, and metasomal sterna largely dark reddish brown (nearly black in many areas), and expansions of protarsi dark brown. Wings orange-yellow except apical margin of forewing and apical and posterior margins of hind wing grayish hyaline (Fig. 17); venation ferruginous to orange-yellow.

Mandible with outer surface shiny, irregularly punctate; vertex with coarse, shallow punctures separated by less than a puncture width, integument between finely imbricate, punctures becoming more shallow toward preoccipital carina; upper gena with irregular punctures separated by finely imbricate integument, remainder of gena and posterior postgena with more regular punctures separated by a puncture width or frequently less, integument otherwise finely imbricate. Mesoscutum anteriorly and medially transversely wrinkled with irregular punctures, such integument blending laterally outside of parapsidal lines and posteriorly to coarsely and contiguously punctate, integument between (where evident) finely imbricate; axillae and mesoscutellum coarsely and contiguously punctate throughout. Dorsal-facing surface of first metasomal tergum imbricate with small punctures nearly contiguous; remaining terga sculptured as on dorsal-facing surface of first metasomal tergum.

Pubescence generally dark fuscous to black except as follows: clypeus apically with dense fringe of long, apically-directed reddish setae (Figs. 18, 19); supraclypeal area with long, numerous, tawny setae, but not entirely obscuring integument; white and black setae arranged on protarsus and apex of protibia as in figure 21; wing setae generally yellow or tawny yellow, without dense cluster of setae forming spot in medial cell (Figs. 17, 20).

*Female*: As for the male except in usual sexual differences and as follows: Total body length 20 mm; forewing length 13.2 mm. Head broader than long (width 5.2 mm, length 3.7 mm); intertorular distance 1.2 torulorbital distance; interocellar distance 2.4 times median ocellar diameter, 1.3 times ocellocular distance; ocelloccipital distance 4.4 times median ocellar diameter; compound eye about twice as long as wide, slightly narrower than gena in profile. Mandible with four teeth; body of mandible short, basal section about as long as or slightly shorter than apical, dentate margin. Labrum rectangular, with apical row of stiff, erect, long setae running along apical margin; apical margin relatively straight. Clypeus without pronounced, medioapical tubercle, margin relatively straight. First flagellomere short, about two-thirds length of second flagellomere; remaining flagellomeres all about twice as long as wide. Procoxae, tibiae, tarsi, and spurs unmodified; pretarsal claws long, curved, simple. Postgradular depressions weaker than in male; terga without transverse ridges, with slight lateral swellings on terga II–IV.

Mandible with outer surface dull, irregularly punctate and microreticulate; clypeus imbricate with coarse, shallow punctures separated by less than a puncture width except along apical border. Mesoscutum anteriorly and medially transversely wrinkled, more weakly so than in male, with irregular punctures, such integument blending laterally and posteriorly to coarsely punctate, punctures separated by less than a puncture width, integument between punctures finely imbricate; axillae and mesoscutellum strongly coarsely punctate, punctures separated by less than a puncture width throughout.

Usual sex differences in setation; pubescence dark fuscous to black except microtrichia on inner surface of mandible dark golden and small dirty white patch on lateral surface of propodeum near metacoxa; clypeus and supraclypeal area not obscured by dense pubescence.

##### Etymology.

The specific epithet is based on the plant generic name *Memecylon* (neuter), and at which the type series was captured.

##### Floral records.

The paratype females were captured at flowers of “*Memecylon umbellatum*” Wall. (Myrtales: Memecylaceae), a *nomen nudum* for what is today recognized by the accepted name *Memecylon lilacinum* Zoll. & Moritz, and not to be confused with *Memecylon umbellatum* Burm.f. in Peninsular India and Sri Lanka.

#### Chalicodoma (Alocanthedon) aterrimum

(Smith)

http://species-id.net/wiki/Chalicodoma_(Alocanthedon)_aterrimum

Figs 35 43

Megachile aterrima Smith 1862: 60.
Chalicodoma aterrimum (Smith); Baker 1993: 223.

##### Additional material.

Indonesia (Sulawesi): 1♂, 1♀, Central Sulawesi, Palolo nr. Palu, vii.1995 [July 1995] (SEMC); 1♂, Central Sulawesi, Sintuwu, SE of Palu, 1.xii.2000 [1 December 2000], I. Steffan-Dewenter (SEMC).

##### Diagnosisn.

Both sexes of this species are easily recognized by the largely hyaline forewings with fuscous apex and dark brown to black venation (Figs. 35, 41). The male resembles that of *Chalicodoma apoicola* in the presence of a dense cluster of setae forming a spot in the medial cell of the forewing (Fig. 39), the outer surface of the protibia distinctly depressed distally (as seen in profile view), and the distinct median emargination of the preapical carina of tergum VI (Fig. 40). It can be separated by the smaller body size (17–19 mm vs. 24.6 mm), the disc of clypeus largely exposed, not covered by dense, appressed setae (Fig. 36), and the shape of protarsi and setation (compare Figs. 14 and 38). As in *Chalicodoma memecylonae* and *Chalicodoma atratiforme*, the clypeal margin of the female is relatively straight, without a medioapical tubercle. In addition to the forewing color (yellow with grayish hyaline apex in those species), the female of *Chalicodoma aterrimum* differs from those species by the sparser and larger punctures on the basal two terga contrasting with the smaller, denser punctures of the remaining segments. In *Chalicodoma memecylonae* and *Chalicodoma atratiforme* the punctures are small and tightly packed on all terga.

#### Chalicodoma (Alocanthedon) atratiforme

(Meade-Waldo) comb. n.

http://species-id.net/wiki/Chalicodoma_(Alocanthedon)_atratiforme

Figs 32–34 44–46

Megachile atratiformis Meade-Waldo 1914: 456.

##### Additional material.

Malaysia (Peninsular): 3♀♀, Pahang, Batu Balai Estate, 18 March 1927, E. Seimund, Ex F.M.S. [Federated Malay States] Museum (NHML); 1♂, 1♀, Negri Sembilan, Gunong Angsi, 2000–2790’ [feet], April 1918, Ex F.M.S. [Federated Malay States] Museum (NHML).

Thailand: 1♀, Uthai Thani Province [western Thailand], Huay Kha Khaeng Wildlife Sanctuary, 15º36’ N, 99º20’ E, 1.xi.1995 [1 November 1995], J. Gazhoul, captured at *Dipterocarpus obtusifolius* Teijsman & Miquel (Dipterocarpaceae), D.B. & M.W. Baker Collection (SEMC); 1♀, 150 n.w. Bangkok, Huay Kha Khaeng, 5.xi.1995 [5 November 1995], J. Ghazoul (NHML).

##### Diagnosisn.

The female of this species can be recognized by the clypeus lacking a medioapical tubercle, short mandibles (Fig. 45), labrum with an apical fringe of erect setae running along relatively straight apical margin, and the yellow wings (Fig. 44). The male can be recognized by the dense setal patch in forewing medial cell, tergum VI with shallow medioapical concavity on preapical carina, protibia with outer, apical surface not depressed, and genitalia as in figures 32–34.

##### Remarks.

It should be noted that Meade-Waldo’s (1914) material from “Middle Tenasserim” is not conspecific with his type (B.M. Type Hym.17a2037) for *Chalicodoma atratiforme* and are actually specimens of *Chalicodoma odontophorum*. Additionally, despite Cockerell’s (1927) assignment of the subspecies *Chalicodoma atratiforme sininsulae* (Cockerell) (Type USNM 70455; ♀; Turtle Island [Koh Tao], Gulf of Siam; *visum*) to Meade-Waldo’s species, his female has nothing whatever to do with *Chalicodoma atratiforme* and is instead a synonym of *Callomegachile (Callomegachile) fulvipenne* (Smith 1879) (**syn. n.**).

##### **Key to Species of** Alocanthedon

**Note:** The female of *Chalicodoma apoicola* is unknown.

**Table:** 

1	Forewing either largely hyaline with fuscous apex (Figs. 35, 41) or entirely dark fuscous (Fig. 10), with dark brown to black venation; preapical carina of male tergum VI with deep medioapical concavity (Figs. 10, 40); outer, apical surface of male protibia distinctly depressed	2
–	Forewing yellow, like parchment, with smoky fuscous or grayish hyaline apex (Figs. 1, 7, 17, 23, 44), with ferruginous to orange-yellow venation; preapical carina of male tergum VI with shallow medioapical concavity (Figs. 6, 22); outer, apical surface of male protibia faintly or not depressed	3
2(1)	Male clypeal disc largely exposed, not obscured by dense, appressed setae (Fig. 36); procoxal spines broad, oblique procoxal lamella long, distance from lamella to outer basal corner of procoxa shorter than lamella length; anterior border of outer surface of protibia with thin fringe of short, erect, black setae; protarsi as in figure 38; terga with faint to absent transverse ridges on non-depressed, postgradular discs; apical margin of second metasomal sternum straight, not produced medially; size moderate (17–22 mm) [Sulawesi]	*Chalicodoma aterrimum* (Smith)
–	Male clypeal surface largely obscured by dense, long, appressed, reddish setae (Fig. 12); procoxal spines more slender, elongate, oblique procoxal lamella short, distance from lamella to outer basal corner of procoxa slightly longer than lamella length; anterior border of outer surface of protibia with dense fringe of long, erect, slightly wavy, black setae; protarsi as in figure 14; terga with strong transverse carinae or ridges on non-depressed, postgradular discs; apical margin of second metasomal sternum with broad, short, medioapical extension; size very large (nearly 25 mm) [Mindanao, Philippines]	*Chalicodoma apoicola* Engel sp. n.
3(1)	Female clypeus with pronounced, erect, medioapical tubercle (Fig. 8); female mandibles elongate (Fig. 8); apical fringe of erect setae on labrum separated from labral apical margin by at least one median ocellar diameter or slightly more, apical margin of labrum medially convex; male clypeus densely covered by long, appressed, apically-directed setae, obscuring integument, face with dense setae outside of antennal toruli tawny or white (Fig. 2); male protarsus as in figure 5 [Thailand, Myanmar]	*Chalicodoma odontophorum* Engel sp. n.
–	Female clypeus without medioapical tubercle; female mandibles short (Figs. 25, 45); apical fringe of erect setae on female labrum running along labral apical margin (separated by less than a median ocellar diameter), apical margin relatively straight; male clypeal disc largely exposed, setae on face outside of antennal toruli largely black or tawny to white only near clypeus (Fig. 18); male protarsus not as in figure 5	4
4(3)	Female mesoscutum with pronounced transverse wrinkling on disc, posteriorly with well-defined coarse punctures separated by a less than a puncture width, punctures irregular, not loosely arranged in transverse series like those in wrinkles (Fig. 25); male forewing without patch of dense setae in forewing medial cell (Figs. 17, 20); male genitalia as in figures 29–31 [Peninsular Malaysia]	*Chalicodoma memecylonae* Engel sp. n.
–	Female mesoscutum with central wrinkling of integument less pronounced, posteriorly with ill-defined, somewhat transverse punctures separated by a puncture width or more and loosely in transverse rows like weak wrinkles on disc (Fig. 46); male forewing with dense patch of black setae in forewing medial cell; male genitalia as in figures 32–34 [Peninsular Malaysia, Thailand]	*Chalicodoma atratiforme* (Meade-Waldo)

## Cladistics

A total of 157 most parsimonious trees (Length = 2275, Consistency Index = 12, Retention Index = 53) were obtained when including *Chalicodoma (A.) memecylonae* in the analysis of the data set of Gonzalez (2008); 58 nodes collapsed in the consensus tree. *Chalicodoma memecylonae* was included in a clade, sister to *Cuspidella*, containing four of the six species of *Callomegachile* used in the analysis: (*Chalicodoma mystaceana* + *Chalicodoma biseta*) + [*Chalicodoma memecylonae* (*Chalicodoma sculpturalis* + *Chalicodoma clotho*)]. The same topology was obtained when deactivating five characters related to parasitism, or excluding one of the outgroup species (i.e., *Dioxys* Lepeletier de Saint Fargeau and Audinet-Serville) or species with missing characters as an attempt to explore other hypotheses of relationships. However, no synapomorphies or high bootstrap values (> 50%) support the placement of *Chalicodoma memecylonae* within *Callomegachile*.

*Alocanthedon* is supported by significant autapomorphies as outlined in the subgeneric description above (*vide supra*). Most features shared between *Alocanthedon* and other *Chalicodoma* subgenera and groups (many of which may be elevated suitably to subgeneric status) are plesiomorphic and are summarized here. The following plesiomorphies are shared between *Chalicodoma memecylonae* and *Callomegachile* in the analyses: first flagellomere shorter than second flagellomere; outer surface of mandible dull, microreticulate to finely punctuate; mesepisternum coarsely punctuate, forming strong rows with distinct shining ridges among them; clypeus of male with sparse setae on basal half, distal half densely covered by setae (integument not visible) [completely covered in two other *Alocanthedon* species as in Fig. 2]; T6 of male without lateral spine on apical margin; male genitalia with rounded or pointed volsella, and penis valve distinctly curved or arched inward. Likewise, *Chalicodoma memecylonae* shares the following plesiomorphies with *Chalicodoma sculpturalis* and *Chalicodoma clotho*, both species of the ‘Eumegachilana’ group: compound eyes strongly diverging ventrally; clypeoantennal distance short, equal to or shorter than vertical diameter of antennal torulus; antennal scape long, at least 1.2 times longer than torulocellar distance; clypeus short, ≥ 3x wider than long; labrum with two types of setae on disc, minute, yellowish, appressed setae, and long (≥ 1 median ocellar diameter); mandible with outer premarginal fimbria reduced or absent; mandible of male with broad, subtriangular, posteriorly-directed process on basal third of inferior border.

## Discussion

The presence of a dense patch of black setae on the forewing of males (except in one species), resembling the dense patch of setae among the submarginal cells of *Thrinchostoma* (Halictidae), is unique among Megachilidae. Likewise, the deep postgenal depression in males having a distinctly protuberant, thick lamella next to the mandible, procoxa with oblique carina or lamella medially on disc, mesobasitarsus with the inner surface concave basally, and the distinctly long, narrow gonostyli of the genitalia are apomorphic characters within *Chalicodoma*. Such characters are distinctive enough to support the separation of those species into a new subgenus. Also, *Alocanthedon* females can be reliably separated from any *Chalicodoma*, particularly those of the subgenus *Callomegachile*, by the clypeal shape (concave to V-shaped on epistomal sulcus basally and medially-projected on distal margin), and the presence of a short, stout seta on the pretarsal claw basally. Females of a few Oriental *Callomegachile* species, such as *Chalicodoma terminalis* (Smith), also have a similar modification in the clypeus but the setae on the pretarsal claws are either normal or they have a different combination of characters (e.g., presence of omaular carina, different shape of labrum, punctation, &c).

Based on the phylogenetic analysis, it may seems best to regard *Chalicodoma memecylonae* as a species group of *Callomegachile* rather than separating it as a new subgenus, despite that no synapomorphies or high bootstrap values (> 50%) supported such relationship in the analysis. Although emphasizing differences is sometimes useful in morphologically homogeneous groups, such as Augochlorini, Euglossini, or Meliponini, the high morphological variation present across Megachilini may lead to an excessive splitting, thus conveying little information regarding relationships. Female characters are usually less variable than male characters in Megachilini, as in the other aforementioned tribes. Given such variation in the male, some species have been separated subgenerically entirely on male secondary sexual characters (such as those found in the male *Chalicodoma memecylonae*) when females are clearly associated with an existing subgenus. For example, the female of *Megachile laeta* Smith, placed in the subgenus *Leptorachina* by Mitchell (1980), is easily assigned to *Megachile* subgenus *Leptorachis* Mitchell based on the mandibular structure and distinctive pubescence of the S6; the male, however, is unlike any other *Leptorachis* in having highly modified front legs that are used to hold the female during mating. Thus, to avoid excessive splitting, it seems more convenient to emphasize the similarities rather than the differences among groups and, although male characters might provide useful phylogenetic information, there is no need to isolate a species into its own subgenus solely on the male morphology.

Female characters (e.g., mandible with outer surface of dull, microreticulate to finely punctuate integument, without cutting edges, mesepisternum coarsely punctuate, forming strong rows with distinct shining ridges among them, and body parallel-sided) also clearly associate *Chalicodoma memecylonae* with *Callomegachile* and its recognition as a separated subgenus may render *Callomegachile* paraphyletic. However, given our limited understanding on the phylogenetic relationships of *Chalicodoma* subgenera as well as of the species groups within *Callomegachile*, such a subgeneric recognition might be desirable for the time being to highlight those autopomorphic characters not found elsewhere in Megachilidae.

A preliminary morphological analysis of Megachilini suggested that *Callomegachile* is likely not monophyletic (Gonzalez 2008). Two of the six species of *Callomegachile* used in the analysis, *Chalicodoma torrida* and *Chalicodoma decemsignata* Radoszkowski, never grouped with the other species of the subgenus in that analysis or when we reanalyzed the same data set including *Chalicodoma memecylonae*. These two distinctive species, as well as *Chalicodoma biseta* and *Chalicodoma clotho* have been subgenerically separated into *Carinula* Michener et al., *Morphella* Pasteels, and *Eumegachilana* Michener, respectively (Michener 2007). However, they have been treated as species groups because of the variation among species within each group and intergradations in some of the main characters that separate them from other *Callomegachile* (Michener 2007). With nearly 100 species described (Ascher and Pickering 2011), *Callomegachile* is the most diverse, morphologically heterogeneous, and widely distributed of all subgenera of *Chalicodoma*. The subgenus is largely tropical, occurring in Sub-Saharan Africa, southern Palaearctic, Australia, and Southeast Asia (Michener 2007). Species vary greatly not only in body size (~8 to 39 mm) but also in the pubescence, the shape of the mandible and labrum, punctation, presence of preoccipital and omaular carinae in both sexes, as well as in the shape of the hidden sterna and genitalia of the male. Without a doubt, given the number of species and astonishing morphological diversity, a detailed phylogenetic analysis of *Callomegachile* is needed. To document the variation in the aforementioned characters, we examined 50 *Callomegachile* species occurring across the distribution range of the subgenus. The variation found among species with unique combination of characters (*n* = 26) is summarized in table 2. The list is not exhaustive as several characters of the hidden sterna and male genitalia were not included. However, we hope to draw more attention to and encourage future phylogenetic studies using these characters. Some of them might prove useful in recognizing natural species groups or inferring floral hosts, such as the presence of capitate hairs on the ventral surfaces of the meso- and metasoma.

**Table 2.** Some morphological characters of certain species of *Chalicodoma* subgenus *Callomegachile* s.l. Female: **1** = labrum triangular; **2** = labrum with distinct fringe of setae basally; **3** = number of mandibular teeth; **4** = elongated mandible; **5** = clypeal carina; **6** = clypeus with slightly concave to V-shaped epistomal sulcus basally; **7** = vertex with a fine, shining longitudinal line from ocelli to posterior margin of vertex; **8** = preoccipital carina; **9** = omaular carina (species with asterisks have omaular carina ventrally only); **10** = pronotal lobe strongly carinate or lamellate; **11** = mesepisternum coarsely punctate, forming strong rows with distinct shining ridges among them; **12** = mesoscutum coarsely punctate, forming strong rows with distinct shining ridges among them; **13** = capitate hairs on ventral surface of mesepisternum and coxae; **14** = pretarsal claws with a seta conspicuously shorter and stouter than the other. Male: **15** = genal concavity; **16** = low juxtamandibular border (not strongly projecting as a lamella as in *Alocanthedon*); **17** = procoxal spine; **18** = modified protarsi; **19** = T6 with strong preapical carina; **20** = T6 with distinct depression above preapical carina; **21** = sterna densely covered by short appressed setae; **22** = volsella. **Dist** = Distribution: O = Oriental, E = Ethiopian; A = Australian; S = Sub-Saharan Africa. Plus (+) and dash (-) symbols indicate presence and absence of a character.

*Characters*																							
*Species*	*1*	*2*	*3*	*4*	*5*	*6*	*7*	*8*	*9*	*10*	*11*	*12*	*13*	*14*	*15*	*16*	*17*	*18*	*19*	*20*	*21*	*22*	*Dist*
*Chalicodoma antinorii* (Gribodo, 1879)	-	-	6	-	-	-	-	-	-	-	-	-	-	-	-	-	+	-	+	-	-	+	E
*Chalicodoma biseta* Vachal, 1903	-	-	4	-	-	-	-	-	-	-	-	+	-	-	+	+	+	+	-	-	+	+	E, S
*Chalicodoma cephalotes* (Smith, 1853)	+	+	3	+	-	-	+	+	+*	+	-	+	+	+	+	+	+	+	-	+	-	+	O
*Chalicodoma chrysorrhoea* (Gerstäcker, 1857)	-	+	4	+	-	-	+	+	+*	+	-	+	+	+	+	+	+	+	+	+	-	+	E
*Chalicodoma devexa* (Vachal, 1903*)*	+	+	4	+	-	-	+	+	-	-	-	+	+	+	+	+	+	+	+	+	+	+	E
*Chalicodoma disjuncta* (Fabricius, 1781)	-	-	4	-	-	-	-	+	+*	+	+	+	+	-	-	-	+	-	+	+	+	+	O
*Chalicodoma excavata* (Cockerell, 1937)	+	+	3	+	-	-	-	+	-	-	-	-	-	+	+	+	+	+	+	+	+	+	E
*Chalicodoma fulvipenne* (Smith, 1879)	-	+	5	-	+	-	-	+	+	+	+	+	+	-	-	-	+	-	-	+	+	+	O
*Chalicodoma grandiceps* (Friese, 1903)	+	+	3	+	-	-	-	+	+*	-	+	+	-	-	+	+	+	+	+	+	+	+	E
*Chalicodoma incisa* (Smith, 1858)	-	+	5	-	-	-	-	+	+	-	+	+	+	-	-	-	-	-	-	+	+	+	O
*Chalicodoma lerma* (Cameron, 1908) *comb. n.*	-	+	4	-	-	-	-	+	+	-	+	+	+	-	-	-	-	-	-	+	+	+	O
*Chalicodoma mephistolica* (Gribodo, 1894)	-	+	4	+	-	-	+	+	+*	+	-	+	+	+	+	+	+	+	+	+	+	+	E
*Chalicodoma mystaceana* Michener, 1962	-	-	5	-	+	-	-	+	-	-	+	+	+	-	-	-	+	+	+	+	+	+	A
*Chalicodoma perniciosa* (Friese, 1903)	+	-	3	+	-	-	-	+	+*	-	+	+	-	-	+	+	+	+	+	+	+	+	E
*Chalicodoma rambutwan* (Cheesman, 1936)	-	+	4	-	-	-	-	-	-	-	-	-	+	-	-	+	+	-	-	-	+	+	O
*Chalicodoma rufipes* (Fabricius, 1781)	+	+	4	+	-	-	-	+	+*	-	+	+	+	-	+	+	+	+	+	+	+	+	E
*Chalicodoma rufiventris* (Guérin-Méneville, 1834)	+	+	3	+	-	-	-	+	+*	-	+	+	+	-	+	+	+	+	+	+	+	+	E
*Chalicodoma simonyi* (Friese, 1903)	+	+	4	+	-	-	-	+	+*	-	-	-	+	+	+	+	+	+	+	+	+	+	E
*Chalicodoma terminalis* (Smith, 1858) *n. comb.*	-	+	5	-	+	+	-	+	-	-	+	+	-	-	-	+	-	-	-	+	-	+	O
‘Carinula’ group																							
*Chalicodoma junodi* Friese, 1904	-	-	4	-	+	-	-	-	-	-	-	-	-	-	-	-	-	-	-	-	-	-	E
*Chalicodoma silverlocki* (Meade-Waldo, 1913)	-	-	5	-	+	-	-	-	-	-	-	-	-	-	-	-	-	-	-	-	-	-	E
*Chalicodoma torrida* (Smith, 1853)	-	-	5	-	+	-	-	-	-	-	-	+	-	-	-	-	-	-	-	-	-	-	E
‘Eumegachilana’ group																							
*Chalicodoma clotho* (Smith, 1861)	+	-	3	+	-	-	+	-	-	-	-	-	-	-	-	-	+	-	+	+	+	+	O
*Chalicodoma monticola* (Smith, 1853)	+	-	3	+	-	-	+	-	-	-	-	-	-	-	-	-	+	-	+	+	+	+	O
*Chalicodoma sculpturalis* (Smith, 1853)	+	-	3	+	-	-	-	+	-	-	-	-	-	-	+	+	+	+	+	+	+	+	O
*Chalicodoma tuberculata* Smith, 1857	+	-	3	+	-	-	+	-	-	-	-	-	+	-	-	-	+	-	-	+	+	+	O

## Supplementary Material

XML Treatment for Alocanthedon
